# Loose suture-related ocular surface inflammation and activation of conjunctiva-associated lymphoid tissue in patients after keratoplasty

**DOI:** 10.1038/s41598-024-61346-2

**Published:** 2024-05-07

**Authors:** Jingrao Wang, Xin Jin, Hao Jin, Di Jin, Hong Zhang

**Affiliations:** 1 Eye Hospital, The First Affiliated Hospital of Harbin Medical University, Key Laboratory of Basic and Clinical Research of Heilongjiang Province, No. 23 Youzheng Road, Harbin, Heilongjiang Province 150001 People’s Republic of China; 2 Departments of Orthopaedics, The First Affiliated Hospital of Harbin Medical University, Harbin, Heilongjiang Province People’s Republic of China

**Keywords:** Keratoplasty, Loose sutures, CALT, Inflammatory cytokines, IVCM, Corneal diseases, Eye manifestations

## Abstract

The purpose of this study is to evaluate loose suture-related inflammation and activation of conjunctiva-associated lymphoid tissue (CALT) in patients after keratoplasty. The patients who were treated with keratoplasty at the First Affiliated Hospital of Harbin Medical University between 2015 and 2022 were recruited into the study. We evaluated the time and location of loose suture development in patients after keratoplasty. In addition, in vivo confocal microscopy was used to evaluate the activation of CALT and the accumulation of inflammatory cells around loose sutures. Meso Scale Discovery assay detection kits were used to evaluate the inflammatory cytokines in the tears of patients before and after the loose suture was removed. In this study, we collected the information from 212 cases (212 eyes) who had PK (126 eyes) and DALK-treated (86 eyes) for corneal transplantation, including 124 males and 88 females, aged 14–84 years old. The average age was 50.65 ± 16.81 years old. Corneal sutures were more prone to loose at 3 months and 6 months after keratoplasty, and the frequent sites were at 5 and 6 o’clock. An increased number of inflammatory cells could be observed around the loose sutures than normal sutures (*P* < 0.001). In CALT, the density of diffuse lymphocytes (*P* < 0.001), follicles (*P* < 0.001), and parafollicular lymphocytes (*P* < 0.001) were higher and the central reflection of the follicles (*P* < 0.001) was stronger when suture loosening happened. The levels of inflammatory cytokines such as IL-1β (*P* = 0.003), IL-8 (*P* = 0.012), and TNF-α (*P* < 0.001) were higher in the tears of the patients with loose sutures. The activation of CALT was partly settled after removing the loose sutures. In conclusion, loose sutures after corneal transplantation can lead to increased infiltration of inflammatory cells, activation of CALT, and increased secretion of inflammatory cytokines in the tears of patients. Regular follow-up to identify and solve the problem in time can avoid suture-related complications.

## Introduction

Keratoplasty is an important means to control the progress of keratopathy, restore corneal transparency, and save vision^1^. Common complications after keratoplasty include corneal graft rejection, secondary glaucoma, postoperative astigmatism, suture loosening, wound leakage, graft ulceration, recurrence of primary disease, etc.^2^. Damaged or loosened corneal sutures have been identified as a major risk factor for graft infection and graft rejection, which may affect the success rate of corneal transplantation^3,4^. However, why loose sutures can cause these complications remains unclear.

In vivo confocal microscopy (IVCM), with a resolution of approximately 1 μm, can be used to observe the subtle changes in the cornea, limbus, meibomian gland, as well as conjunctiva^5–10^. In our previous study, we found that conjunctival-associated lymphoid tissue (CALT) was activated in keratitis, diabetes, and MGD, and may predict the prognosis of these diseases using IVCM^10–12^. Based on the above studies, we hope to evaluate the inflammatory response and find out whether loose sutures can cause increased corneal rejection with IVCM.

The current study was performed to evaluate potential risk factors, prone time, and position for suture loosening development. Also, we evaluated the inflammatory cells around the loose suture, the activation of CALT, and the level of inflammatory cytokines in tears.

## Results

### Clinical data of patients who underwent keratoplasty

**Figure 1:**
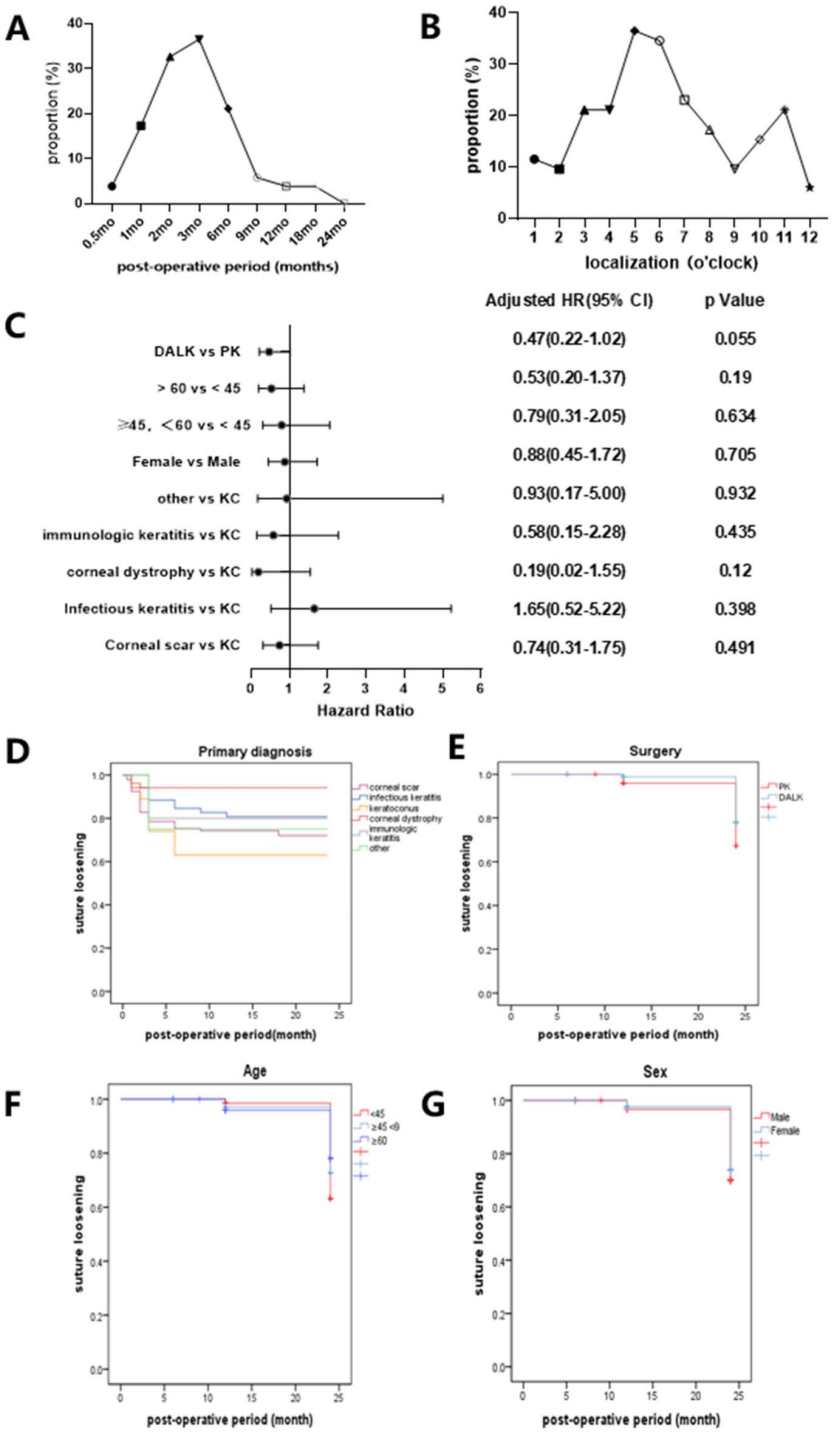
The condition of suture loosening after keratoplasty. (**A**,**B**) Line chart of the proportion of suture loosening in different months and localization after keratoplasty. (**C**) Forest plots displaying multivariate analysis and hazard ratios associated with suture loosening. (**D**–**G**) Kaplan–Meier plots of freedom from suture loosening in patients with different primary diagnoses, surgery techniques, ages, and sex. DALK, deep anterior lamellar keratoplasty; PK, penetrating keratoplasty; KC, keratoconus.

Descriptive statistics for the post-operative patients are described in detail in Table 1. We collected the information from 212 cases (212 eyes) who had PK (126 eyes) and DALK-treated (86 eyes) for corneal transplantation, including 124 males and 88 females, the average age was 50.65 ± 16.81 years old, and 58.5% of the patients were men. Figure 1A illustrated that suture loosening most frequently occurs at 3 months (32.7%) and 6 months (36.5%) after the keratoplasty. Figure 1B figured out that suture loosening most frequently occurs at 5 (36.5%) and 6 (34.6%) o’clock. To evaluate potential risk factors for suture loosening development, we found no significant difference in surgery (*P* = 0.055), age (*P* = 0.19, 0.634), sex (*P* = 0.705) as well as primary diagnosis (*P* = 0.932, 0.435, 0.12, 0.398, and 0.491) (Fig. 1C). Kaplan–Meier survival curves showed that there is no statistically significant impact of primary diagnosis, surgery, age, and sex on the timing of suture loosening. (*P* > 0.05) (Fig. 1D–G).

**Table 1:** Descriptive statistics for the post-operative patients.

Suture loosening, n = 52 (24.5%)	Normal, n = 160 (75.5%)
Surgery	Surgery
PK, n = 36 (69.2%)	PK, n = 90 (56.3%)
DALK, n = 16 (30.8%)	DALK, n = 70 (43.7%)
Sex	Sex
Male, n = 33 (63.5%)	Male, n = 91 (56.9%)
Female, n = 19 (36.5%)	Female, n = 69 (43.1%)
Age	Age
< 45, n = 13(25.0%)	< 45, n = 46(28.8%)
≥ 45, < 60, n = 25(48.1%)	≥ 45, < 60, n = 60(37.5%)
≥ 60, n = 14(26.9%)	≥ 60, n = 54(33.8%)
Primary diagnosis	Primary diagnosis
Corneal scar, n = 26(50.0%)	Corneal scar, n = 67(41.9%)
Infectious keratitis, n = 10(19.2%)	Infectious keratitis, n = 42(26.3%)
Keratoconus, n = 10(19.2%)	Keratoconus, n = 17(10.6%)
Corneal dystrophy, n = 1(1.9%)	Corneal dystrophy, n = 16(10.0%)
Immunologic keratitis, n = 3(5.8%)	Immunologic keratitis, n = 12(7.5%)
Other, n = 2(3.8%)	Other, n = 6(3.8%)

PK, penetrating keratoplasty; DALK, deep anterior lamellar keratoplasty.

### Cornea with loose sutures can be observed by corneal fluorescence staining, AS-OCT, and IVCM

**Figure 2:**
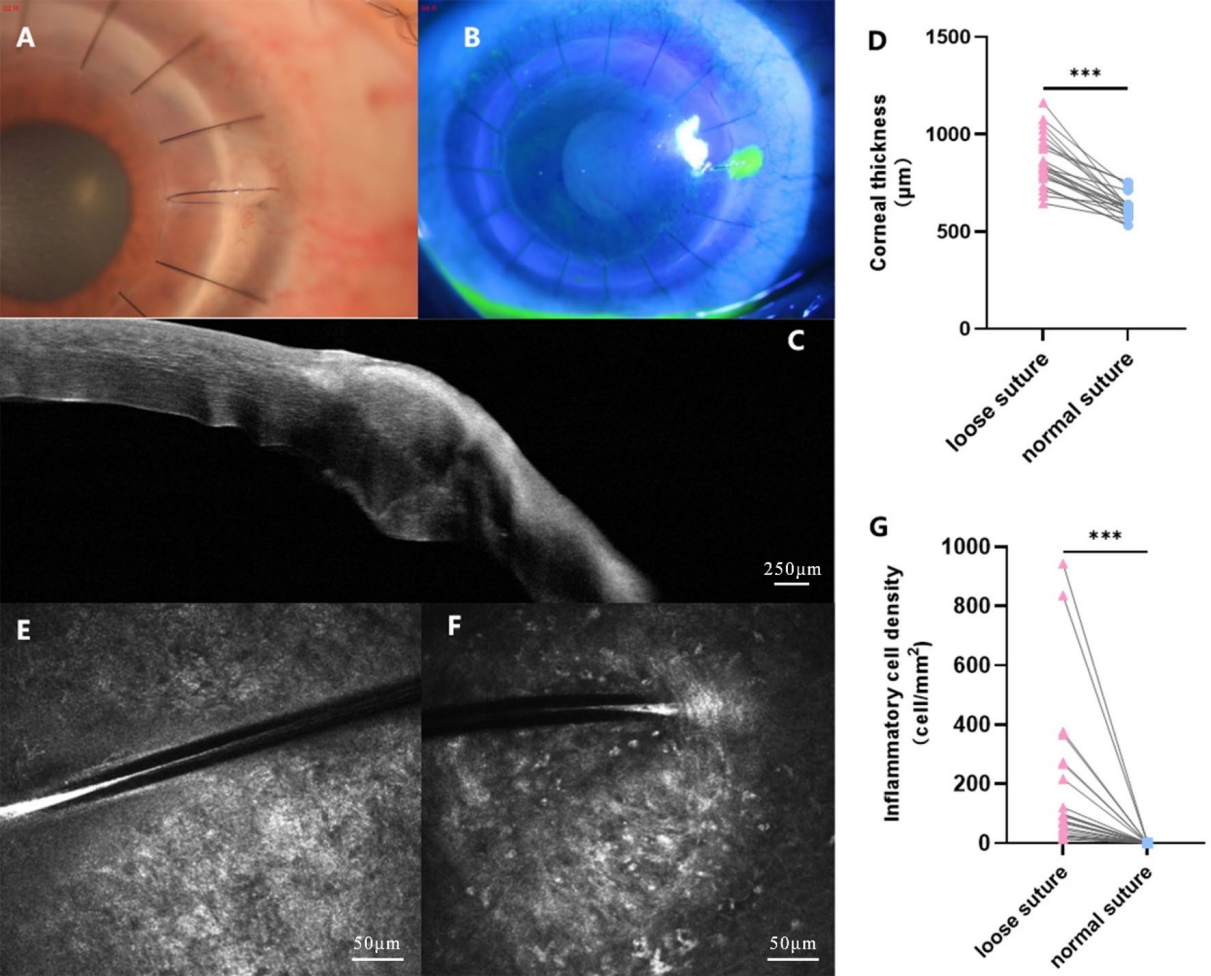
Cornea with loose sutures can be observed by corneal fluorescence staining, AS-OCT, and IVCM. (**A**) Loose sutures can be found from the anterior segment photography (taken by Xin Jin); (**B**) Corneal fluorescence staining was positive around loose sutures (taken by Xin Jin); (**C**,**D**) The cornea was swollen around the loose sutures by AS-OCT; (**E**) No inflammatory cell infiltration was found beside the normal sutures by IVCM; (**F**,**G**) Inflammatory cell infiltration was found beside the loose sutures by IVCM. ***P < 0.001.

Figure 2A,B showed that the loose sutures were stained with fluorescein, indicating that the corneal epithelial cells were destroyed around the loose sutures. AS-OCT showed that the cornea was swollen around the loose sutures (*P* < 0.001) (Fig. 2C,D). IVCM showed that the density of inflammatory cells around the loose sutures was significantly higher than that around normal sutures (75 cells/mm^2^ [Q25–Q75: 25–267 cells/mm^2^] vs. 0 cells/mm^2^ [Q25–Q75: 0–0 cells/mm^2^], *P* < 0.001) (Fig. 2E–G).

### Analysis of the change in CALT-related parameters

**Figure 3:**
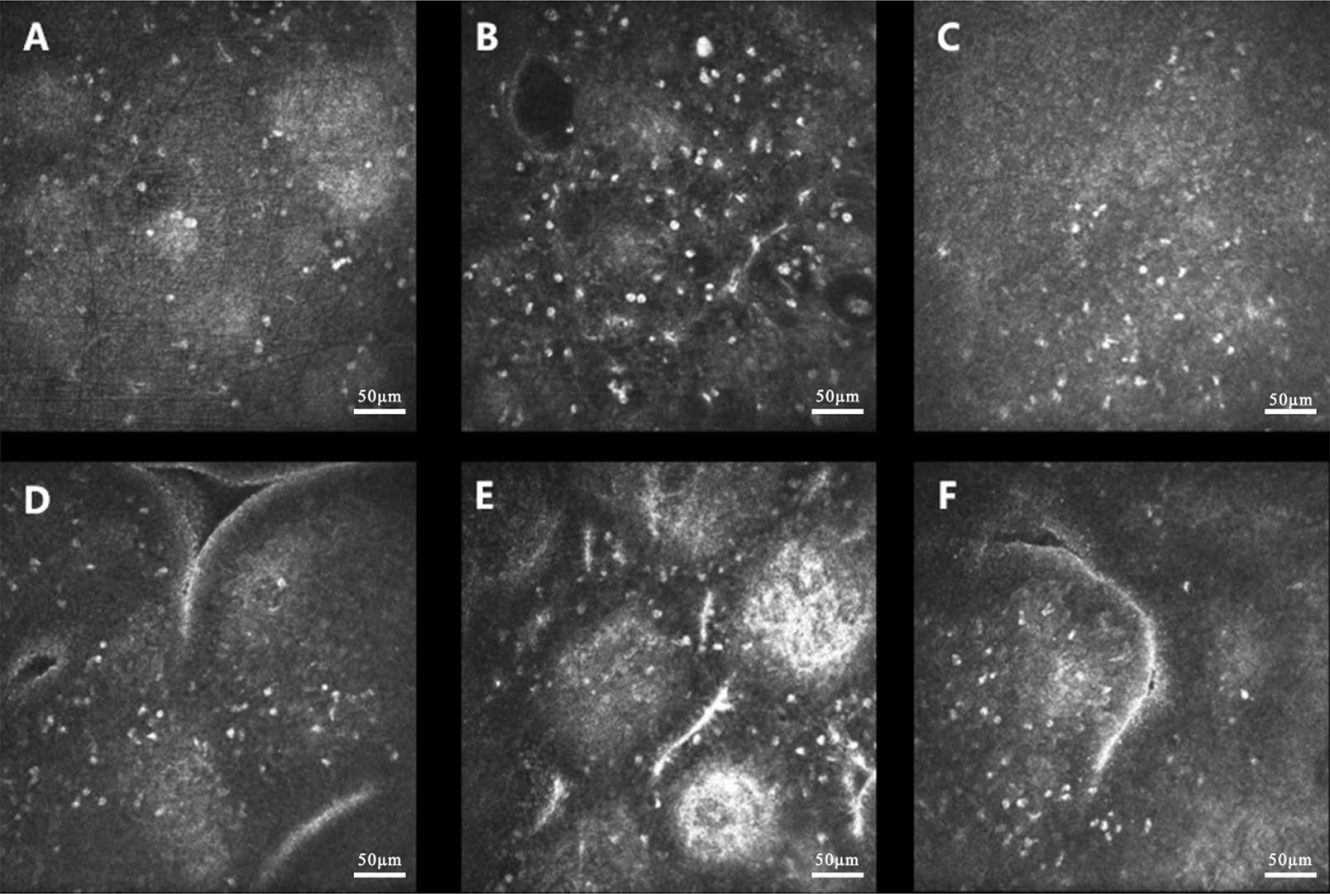
(**A**–**C**) IVCM images of diffuse lymphoid tissue in one patient after keratopathy before suture loosening, during suture loosening, and one week after the removal of the loose sutures; (**D**–**F**) IVCM images of lymphoid follicles in one patient after keratopathy before suture loosening, during suture loosening, and one week after the removal of the loose sutures.

**Figure 4:**
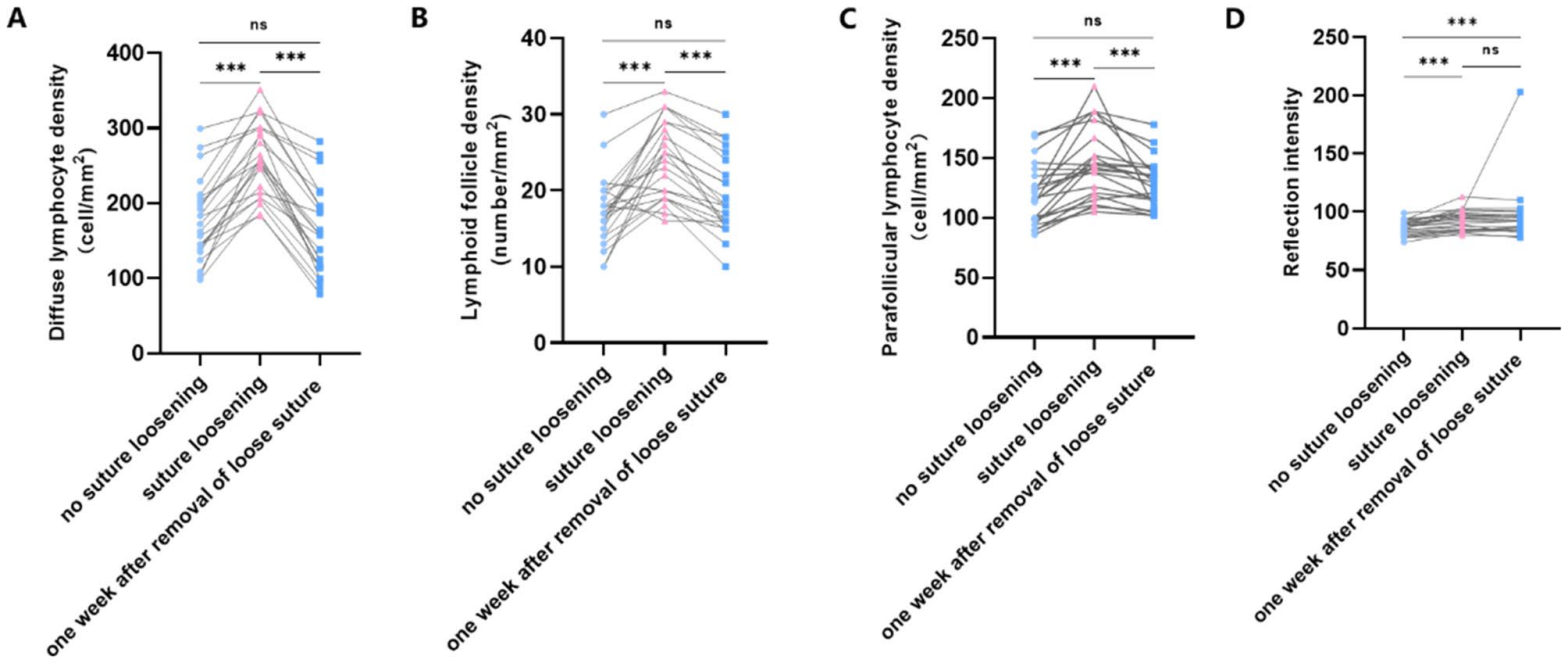
Analysis of the change in CALT-related parameters. Changes of the diffuse lymphocyte density (**A**), lymphoid follicle density (**B**), parafollicular lymphocyte density (**C**), and the central reflection of the follicles (**D**) in each period. ***P < 0.001.

Compared with no suture loosening, the diffuse lymphocyte density in patients increased significantly when loosening occurred (*P* < 0.001). Furthermore, follicle-related parameters including lymphoid follicle density (*P* < 0.001), parafollicular lymphocyte density (*P* < 0.001), and follicular center reflection intensity (*P* < 0.001) was also increased. One week after the removal of the loose sutures, certain CALT parameters including the diffuse lymphocyte density (*P* < 0.001), lymphoid follicle density (*P* < 0.001), and parafollicular lymphocyte density (*P* = 0.004) recovered from suture loosening occurred. However, there was no significant difference in follicular center reflection intensity (*P* > 0.05) (Figs. 3, 4 and Table 2).

**Table 2:** The change in CALT-related parameters.

CALT	No suture loosening	Suture loosening	One week after the removal of loose suture	*P* value
Diffuse lymphocyte density (cells/mm^2^)	177.04 ± 54.05	259.91 ± 47.97	162.04 ± 57.85	*P* < 0.001
Lymphoid follicle density (follicles/mm^2^)	17.26 ± 4.59	24.22 ± 5.09	19.35 ± 5.14	*P* < 0.001
Parafollicular lymphocyte density (cells/mm^2^)	118.35 ± 24.90	145.17 ± 30.02	127.96 ± 20.62	*P* < 0.001
Follicular center reflection intensity	88 [Q25–Q75: 82–91]	94 [Q25–Q75: 85–99]	93 [Q25–Q75: 84–98]	*P* < 0.001

Data are shown as mean ± standard deviation or median (Q25–Q75).

### The expression of IL-1β, IL-8, and TNF-α was decreased in the tears of patients after the removal of loose sutures

**Figure 5:**
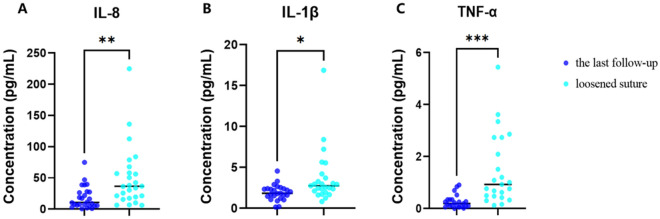
The expression of IL-1β, IL-8, and TNF-α was decreased in the tears of patients after the removal of loose sutures. (**A**) IL-8: *P* = 0.003, (**B**) IL-1β: *P* = 0.012, (**C**) TNF-α: *P* < 0.001 decreased significantly in patients one week after the removal of loose sutures. ***P < 0.001, **P < 0.01, *P < 0.05.

Compared to when the sutures loosened, 1 week after their removal, the inflammatory cytokines including IL-1β (*P* = 0.012), IL-8 (*P* = 0.003), and TNF-α (*P* < 0.001) in the patient’s tears decreased significantly (Fig. 5).

## Discussion

Our results showed that there was no significant correlation between suture loosening and gender, age, primary corneal disease, and surgical procedures. Suture loosening most frequently occurs at 3 months (32.7%) and 6 months (36.5%) after the keratoplasty, and the location of 5 (36.5%) o’clock and 6 (34.6%) o’clock. When loose suture occurred, the inflammatory cells around the loose sutures increased significantly and the inflammatory cytokines in the tears of patients, including IL-8, IL-1β, and TNF-α, increased as well. CALT was also significantly activated with loose sutures. One week after the loose sutures were removed, the inflammatory cytokines decreased and CALT activation was also partly settled.

According to our results, 24.5% of patients had loose sutures during follow-up after keratopathy, which is similar to Dana’s results^13^. Corneal sutures were more prone to loose at the 5 o’clock and 6 o’clock positions. The location of our study is different from others. Reza Dana^13^ reported that the locations of eroded sutures were mainly superior (53%), and 29% of patients had loose sutures during follow-up in a retrospective analysis. Fong^4^ found that the suture-related microbial infection is mainly located in the palpebral fissure. Recently, Sepehr^14^ found loose sutures after DALK for keratoconus were located superiorly in 24.7%, inferiorly in 27.1%, temporally in 28.0%, and nasally in 20.2% of the episodes. Li^15^ also found suture loosening after keratoplasty mainly occurred in the inferior cornea (62%). The reason for these phenomena may be because the eyelid partially covers the upper cornea, which can partially resist intraocular pressure and the corneal self-elastic tension on the upper graft, thereby reducing the rate of loosening of the upper sutures^15^. Also, mechanical trauma caused by blinking leads to more vigorous cell renewal and wound contracture, and therefore more prone to suture loosening^16^.

Theoretically, sutures can become loose in three ways: (1) The sutures are tight at first but later become loose as corneal edema subsides. (2) The epithelial barrier function is incomplete due to sutures, and microorganisms can penetrate and cause matrix infiltration. The resulting population of white blood cells softens the matrix tissue, followed by matrix loss, loss of tension of the sutures, and further erosion to the surface. (3) Due to the biodegradation of nylon, the suture may become loose in the later stage^17–20^. Nylon corneal sutures are related to infectious keratitis, and in some cases may cause endophthalmitis. Studies have shown that there are bacteria and biofilms in the corneal sutures^21^. Once the corneal sutures are exposed or develop keratitis, the sutures should be removed as soon as possible.

Loose sutures can cause corneal sterile infiltrates (9.4%)^22^, and imminent wound dehiscence (8.3%)^23^. Also, loose sutures are a major predisposing factor for corneal graft ulcers because the exposed end of a suture can trap mucus or debris, acting as a nidus for the colonization of microorganisms and eventually cause microbial keratitis (1–3.3%)^4,22–25^, which is a leading cause of corneal graft rejection and graft failure^4^. In our study increased number of inflammatory cells can be observed around the loose sutures than normal sutures by IVCM; the expression of IL-1β, IL-8, and TNF-α were increased in the tears by MSD assay detection kits. CALT is mainly located in the lamina propria containing lymphoid layer (mainly T cells and IgA-secreting plasma cells), lymphoid follicles (mainly B cells, T cells, macrophages, and dendritic cells), and crypt-associated lymphoid structures^26,27^. There is growing evidence that CALT is activated in many inflammatory ocular diseases, such as infectious keratitis, dry eye, diabetes, and MGD^9–11,27^. In our study, we also found that the CALT was activated when the patients were suffering from suture loosening, which was consistent with the increase of inflammatory cells around the loose suture. One week after the removal of the loose suture, activation of the CALT was partly recovered. Our team previously found one patient who didn’t remove the loose suture in time and eventually developed fungal keratitis under the loose suture 4 weeks after the presence of loose suture^28^.

The above results indicate that an inflammatory response can be triggered by loose suture, and the inflammation subsides after suture removal. But at the same time, we should also pay attention to the problem of wound dehiscence after the removal of sutures, and re-suture of the corneal graft may be required depending on clinical presentation^29^. Regular follow-up to identify and solve the problem in time can avoid suture-related complications.

## Methods

### Participants

This is a single-center study that evaluated all patient charts with a corneal transplantation surgery between 2015 and June 2022 at the First Affiliated Hospital of Harbin Medical University. All patients gave informed consent to the study content. Instruct the patient to come for follow-up observation at the clinic after half a month, one month, three months, and six months following corneal transplantation surgery. Additionally, instruct them to return to the clinic one week after removal of the loose sutures for further follow-up examination. Inclusion criteria: patients diagnosed with corneal scar, infectious keratitis, keratoconus, corneal dystrophy, immunologic keratitis by an experienced physician and who can tolerate keratoplasty surgery; Exclusion criteria: (1) Persons > 85 years old; (2) uncontrolled hypertension or poor response to drug treatment; (3) Patients with unstable blood glucose control and fasting blood glucose still higher than 7.0 mmol/L after drug treatment; (4) An active infection of the eye.

### Corneal transplant surgery

After routine disinfection, the upper and lower rectus muscles were fixed with suture, and the conjunctival capsule was rinsed with povidone iodine. PKs were performed using standard techniques. The donor was excised much larger diameter (0.25 mm) than the recipient using a manual trephine system. Intraocular viscoelastic injections were applied to protect the corneal endothelium of grafts. The graft was secured to the host bed using 16 interrupted 10–0 nylon non-absorbable surgical sutures (Johnson & Johnson). At the end of each operation, the anterior chamber was restored by saline injection and the watertightness of corneal wound was carefully checked. In DALK procedure, the diameter range of the host bed was similar to that in PK. After a partial trephination of approximately 50% of the host corneal stroma and manual stripping of the superficial stromal layer, pneumatic pressure was used to detach the Descemet’s membrane (DM) from the deep stroma by injecting sterile air into the latent space between these two adjacent corneal microstructures. Air injection would produce a dome-shaped bubble that could be seen under the surgical microscope. Corneal stromal tissue above the “bubble” was manually dissected with scissors and a spatula until a complete exposure of DM underneath was achieved. The same-size donor without DM and endothelium was then sutured to the recipient using a 16-bite interrupted suturing technique. All corneal transplant operations are performed by the same experienced professor.

### The collection of tears

The patient’s tear samples were collected at the time of suture loosening and one week after the removal of the loose sutures. The qualitative filter paper was cut to a size of 0.5 mm × 20 mm. The patient was instructed to look upward, and the filter paper was placed in 1/3 of the lower eyelid (to avoid contact with cornea and bulbar conjunctiva), then the patients were instructed to look forward, and after 5 min, the filter paper was removed and immediately stored in the − 80 °C.

### MSD assay detection kits

The MSD assay detection kits were used to evaluate the inflammatory factors in the tears of the patients. Cytokine secretion was evaluated by V-PLEX Human Cytokine 30-Plex Kit with measurements by Meso QuickPlex SQ120 (Meso Scale Discovery, Rockville, MD, USA). After drawing the standard curve, the antibody to be tested was diluted. According to the experimental configuration, a 50 ul tear sample (the time on suture loosening and the last follow-up) was added to the corresponding hole, and the antibody was detected, and shaken at room temperature for 2 h. The MSD plate (Meso Scale Discovery, Rockville, MD, USA) was cleaned, and 25ul antibody was added to each hole and shaken at room temperature for 2 h. Clean the MSD board, add 150ul of prepared board reading liquid to each hole, and check it on the Meso QuickPlex SQ120.

### AS-OCT

23 eyes in 52 patients with loose sutures were imaged by AngioVue® OCT angiography (OCTA) (Optovue, Inc., Fremont, CA, USA) (Optical resolution: vertical 5 μm, horizontal 15 μm; Scan condition: Cornea Cross Line model) to evaluate corneal thickness around the loose sutures compared to normal sutures.

### IVCM

23 eyes in 52 patients with loose sutures were imaged by IVCM (Heidelberg Retinal Tomograph 3 with the Rostock Cornea Module; Heidelberg Engineering GmbH, Heidelberg, German) (Resolution: 1 μm) to evaluate whether inflammatory cells were visible around loose sutures, IVCM scans were also performed on the normal sutures of the same patient. The detection method of CALT can refer to the previous research^5,10^.

### Statistical analysis

Data was analyzed using the SPSS software version 22.0 (SPSS Inc., IBM, Chicago, IL, USA) and GraphPad Prism 9 for Windows (version 9.0.0; GraphPad Software). Group comparison was conducted according to age, sex, surgical procedure, primary disease, and other influencing factors, and multivariate analysis was adopted. Survival curves were analyzed by the Kaplan–Meier method. Paired sample t-test and Wilcoxon signed rank test were performed for corneal thickness, the number of inflammatory cells, and the contents of IL-1β, IL-8, and TNF-α in the tear. A repeated measure analysis of variance (ANOVA) with Bonferroni post-hoc, or Friedman’s ANOVA with Wilcoxen Signed Rank tests follow-up, according to data distribution, were used to assess CALT-related parameters in patients before suture loosening, during suture loosening, and one week after removal of the loose suture. *P* < 0.05 was statistically significant.

### Ethics approval

The study was approved by the Ethics committee of the First Affiliated Hospital of Harbin Medical University (IRB-AF/SC-05/04.0). The study obtained written informed consent from the participants. Published studies comply with the Helsinki Declaration of the World Medical Association.

## Data Availability

All data is raw, unmodified or modified, and is publicly available with the permission of the author Hong Zhang (zhanghong@hrbmu.edu.cn).
